# Mindset modification of community pharmacists in a collaborative relationship between a major hospital and neighboring community pharmacies: a qualitative study

**DOI:** 10.1186/s12913-019-4253-4

**Published:** 2019-07-15

**Authors:** Miwa Hinata, Kikuko Miyazaki, Hiroshi Okada, Takeo Nakayama

**Affiliations:** 1 Department of Hospital Pharmaceutics, School of Pharmacy, Showa University, 1-5-8 Hatanodai Shinagawa-ku, Tokyo, 142-8555 Japan; 2 Department of Health Informatics, Kyoto University School of Public Health, Yoshida-Konoe-cho, Sakyo-ku, Kyoto, 606-8501 Japan

**Keywords:** Qualitative study, Pharmacy, Collaboration

## Abstract

**Background:**

Patient information sharing between hospitals and community pharmacies is generally insufficient. Since August 2013, the pharmacy department of Kyoto University Hospital has initiated and mediated a collaborative relationship between physicians and neighboring community pharmacies (e.g., sharing outpatient blood test results, holding regular meetings among professionals, delivery of tracing reports from community pharmacists to physicians about outpatients).

**Methods:**

This study describes how community pharmacists have developed as a result of this professional collaboration (known as the “Kyoto University Hospital model”) and attempts to grasp its current situation through interviews with pharmacists. The authors conducted semi-structured individual interviews with community pharmacists between June and December 2014. The interview data were analyzed using the constant comparative method.

**Results:**

Twenty-one pharmacists working for 11 neighboring community pharmacies were interviewed, at which point theoretical saturation was achieved. The mean interview time was about 50 min. Among the participants, there were 15 women and 6 men; 10 were pharmacist managers and 11 were staff pharmacists. Through the analysis of the interview data, 13 categories were generated from 32 concepts. The results indicated that, through the Kyoto University Hospital model, community pharmacists shifted from a “Mindset of being the hospital’s subcontractor” to “Being motivated to participate in team care.” Specifically, their professional attitude shifted in a positive direction to “Being motivated to participate in team care”, which was a departure from their previous feelings of inadequacy, related to their “Mindset of being the hospital’s subcontractor” and how “Barrier to medicine counseling”.

**Conclusions:**

Under the Kyoto University Hospital Model, hospital pharmacists encouraged active collaboration between physicians, hospital pharmacies, and community pharmacists by cultivating face-to-face relationships. This in turn helped community pharmacists become more conscious of their expert status, and thereby participate actively in patients’ treatment.

## Background

Recently, in developed countries with rapidly aging populations, securing the effectiveness and safety of medication therapy for outpatients has become a major issue. To this end, the 2011 Good Pharmacy Practice guidelines (a joint initiative by the International Pharmaceutical Federation and World Health Organization) [1] proposed that pharmacists should forge therapeutic collaborative partnerships based on mutual trust and confidence with other health professionals, particularly physicians, in all matters relating to pharmacotherapy. However, in Japan, interprofessional collaboration between hospitals and community pharmacies for outpatients remains insufficient [2].

The separation of prescribing and dispensing, also called dispensing separation, has been practiced in Japan since 1956 [3]. As a result, the role of community pharmacies as a means of obtaining medication, as opposed to hospitals dispensing medicines directly to outpatients, has been widely promoted. By 2015, about 80% of prescriptions in Japan were sent to community pharmacies [4]. Dispensing separation has the advantage that pharmacists can manage the duplication and interaction of patients’ prescribed medicines multiple hospitals, prescription drug-related problems and so on. At present, however, Japan does not make full use of the potential advantages of this system [5]. One major barrier is inadequate information-sharing between medical institutions [2]. According to a survey by the Pharmaceuticals and Medical Devices Agency (PMDA) in 2014, 9.6% of hospitals indicated that they provided information on all patients to insured pharmacies [6]. This is partly because in its regulation of prescriptions, the Doctor Act has no requirement for the provision of patient clinical information (e.g. blood test values and diagnoses) in a prescription [7]. Therefore, if the patient does not present their information to a pharmacist, they must rely on limited information, making it difficult to confirm the appropriate dosage (which requires blood test results) and monitor adverse drug reactions [8]. Meanwhile, patients’ drug-related problems or medication adherence may be recorded at community pharmacies, but this information is rarely shared with hospital doctors or pharmacists.

A tracing report system was implemented in 2012, involving the feedback of information recorded at the community pharmacy to the prescribing doctor. When sending a tracing report, community pharmacies can calculate the insurance score as a medication information provision fee. However, tracing reports were issued for only 0.04% of the total number of prescriptions in 2014 [9].

In an effort to improve this situation, in August 2013, the Kyoto University Medical School Hospital (hereinafter, Kyoto University Hospital) established a system of medical collaboration between physicians and neighboring community pharmacies, mediated by the hospital pharmacy department [10]. The collaboration involved (1) regular meetings at the hospital pharmacy department for 11 neighboring community pharmacies that receive a large number of prescriptions from Kyoto University Hospital; (2) the use of tracing reports; and (3) the sharing of information on 13 blood test items (starting from October 2013) between hospitals and community pharmacies. A total of 124 tracing reports were submitted from the pharmacy to the prescribing physician in the first eight months of the collaboration [10]. Overall, Kyoto University Hospital has deployed a multifaceted approach to promote patient information-sharing, referred to as the “Kyoto University Hospital model.” However, it remains unclear what form of conscious change has occurred in community pharmacists as a result of using this style of collaboration. Clarifying community pharmacists’ perceptions of this model might provide useful information to encourage coordination between major hospitals and nearby community pharmacies in other parts of the country. Therefore, this study examined the change in mindset of neighboring community pharmacists as they participated in the Kyoto University Hospital model of collaboration.

## Method

This was a qualitative interview study. Subjects were community pharmacists from the 11 community pharmacies around Kyoto University Hospital. Participants were eligible for inclusion if they were pharmacists who (1) had participated in one of the regular collaboration meetings held by the Kyoto University Hospital Pharmacy Department; (2) had worked in one of the 11 neighboring pharmacies for at least one year (to enable comparison with the situation one year ago); and 3) had at least three years’ work and dispensing experience. We used purposive sampling to select participants, aiming to obtain at least one pharmacist from each of the pharmacies, to ensure a balanced response. The manager of each pharmacy was asked to introduce the authors to working pharmacists, so they could be recruited directly. This recruitment procedure, which was conducted concurrently with data collection and analysis, continued until theoretical saturation was reached (i.e., no new knowledge was obtained through continuing the process).

The data collection was carried out via semi-structured, individual, in-depth interviews using an interview guide (Table 1).

**Table 1:** Interview guide

- What do you think about the meetings with the Kyoto University Hospital and the description of the blood test values on prescriptions?	
- What has changed compared to before blood test values were written on prescriptions?	
- How has the relation between the doctor or patient changed compared to before blood test values were written on the prescriptions?	
- Are there any problems in sharing patient information with doctors?	
- In what situations do you think it is easy to provide information to doctors?	
- Why is it difficult for doctors to share information?	

In-depth interviews were selected because the authors needed frank appraisals of the Kyoto University Hospital model. The interviews were conducted at the pharmacy where each participant worked, and was audio-recorded with participants’ consent. Each interview was conducted by the first author (MH), an expert interviewer with experience working as a community pharmacist. As background, the authors administered a questionnaire asking participants about their participation status in the collaboration.

A verbatim record of each interview was prepared from the recordings and used as language data for analysis. The data were analyzed using the constant comparative method, a type of thematic analysis considered particularly accurate for explaining and predicting human behavior [11], and therefore suitable for clarifying how community pharmacists share information with hospitals and reflect on their work. An analysis worksheet for the modified grounded theory approach (M-GTA) [11] was used to analyze the data.

The analysis procedure was as follows. We analyzed the data inductively by checking the analysis theme while in contact with the verbatim records. We then developed concepts from each part of the text we focused on (called “variations”), corresponding to the analysis theme. The concept creation and naming process was recorded in theoretical memos. Once a concept was nearly completed, the textual data was examined again to determine if there was an example of an opposite concept. When an opposite case existed, the concept was finalized; if no opposite existed, this was noted in the theoretical memo of the analysis worksheet. Concept development and opposite case-finding were repeated to generate further concepts and categories, and the relationships between the concepts and categories were examined and recorded in theoretical memos. The findings generated through this process was reviewed by a supervisor (KM) and multiple analysts, and their findings were triangulated [12].

This research was approved by the Kyoto University ethics committee of medical doctors (approval number 1094, E2396). The purpose of the study and privacy protection was explained to all research collaborators and cooperating organizations, and both written and verbal consent was obtained from participants.

## Results

### Basic characteristics

From June to December 2014, interviews were conducted with 21 pharmacists working for the 11 neighboring community pharmacies. Theoretical saturation, when no new knowledge is obtained through further investigation, was reached by the 19th participant, and we conducted two more interviews to confirm this. The mean time for interviews was 50 min. The participants were 15 women and six men; 10 were pharmacist managers and 11 were staff pharmacists. Their median practice experience was 14.5 years, and 17 of the 21 participants had participated in the regular meetings three or more times (nine meetings were held between August 2013 and December 2014). Eight participants had not submitted a tracing report (Table 2).

**Table 2:** Collaboration status and characteristics of participants

Sex	Man	6
	Woman	15
Job Category	Staff	11
	Manager	10
Experience year (median)		14.5(4–45)
Participate in meeting (times)	0	2
	1	2
	2	0
	≥3	17
Submitting tracing report (times)	0	8
	1	2
	2	4
	≥3	7

Through the analysis of the interview data, 13 categories were generated from 32 concepts (Table 3).

**Table 3:** Concepts and categories

Before collaboration	
【Barrier to medicine counseling】 (Patient who does not want to talk) (Medication counseling without information)	If the patient does not want to talk about his own illness at the community pharmacy, it was impossible to ensure reliable pharmaceutical management (adverse events and proper dose confirmation), and the community pharmacist had to provide medicine while grappling with anxiety.
【Mindset of being the hospital’s subcontractor】 (Patient who cannot be honest with their doctor) (Minimal instruction acceptance) (Sparse involvement with hospitals) (One-way meetings) (Unapproachable university hospital)	Community pharmacists became passive because they felt like they were the hospitals’ subcontractors rather than equals. The meetings over the past year had not provided opportunities to exchange opinions and, as a result, they had a basically non-existent relationship with the hospital. Community pharmacists often hesitated to convey a trivial problem with patient treatment to the attention of hospital doctors or the pharmacy department.
Starting the collaboration	
**【**Hospital pharmacist’s visit**】**	Community pharmacists were very surprised that the director of Kyoto University Hospital pharmacy visited their community pharmacies. This interaction helped close the gap between them and hospital pharmacists.
**【**The beginning of the medical collaboration**】**	Hospital pharmacists actively encouraged community pharmacists, and they in turn were motivated to provide treatment support for hospital patients.
**【**Confrontation with blood test data**】**	Community pharmacists had minimal experience in pharmacological management via blood test values and felt that they could not master it.
**【**Puzzled by the tracing report**】** (Difficulty of document transmission) (Speed of doubt inquiry)	Community pharmacists could provide the tracing report to the hospital doctor without knowing what to report. Furthermore, they felt it was faster to call the doctors by telephone than faxing the tracing report to them.
After collaboration	
**【**Recognition of roles**】** (Friendship with hospital)	Community pharmacists participated in a meeting hosted by a hospital pharmacy where they shared their therapeutic policies with patients. This helped them become aware of their role in terms of providing information to the patient about the treatment prescribed by the doctor.
**【**Interaction with doctors**】** (Understanding of prescription intention)	Community pharmacists interacted directly with the hospital doctors at the study meetings, which helped to familiarize them with the doctors’ personalities. This made it easier to consult with doctors when they noticed prescription problems.
【Awareness of their responsibility】 (Indicator of disease condition) (Accurate medication consultation) (Place of OJT)	Community pharmacists felt a sense of responsibility and confusion about having to corroborate the prescription with blood test values, which they had hardly used before. However, by applying the knowledge gained at the blood test study meeting, their pharmacy became a place for on-the-job training (OJT).
**【**Change to proper administration of medication dose**】** (Confirmation of adverse events using blood test values)	Community pharmacists could consider inquiries to hospital doctors after checking the prescriptions and taking into consideration factors such as renal and liver function. Furthermore, it became possible for them to deliver drugs after confirming adverse events.
**【**Support of the hospital pharmacists**】** (Anxiety about future policy change) (Willingness to improve skills)	When a community pharmacist reported patients’ drug adherence to a doctor, they gained the support of hospital pharmacists; this helped them submit tracing reports without anxiety.
**【**Active use of the tracing report**】** (Giving adherence reports to doctor)	If a community pharmacist noticed a therapeutic problem in a patient, no matter how small, they submitted a tracing report.
**【**Being motivated to participate in team care**】** (Pharmacist trusted by patient)	Community pharmacists had more opportunities to receive gratitude from physicians and patients. As a result, they began feeling a sense of responsibility and satisfaction, and wanted to be increasingly involved in patients’ treatment.

Note: 【Category】 and (concept)

Storyline constructed using 【Category】and (concept)

Before the collaboration, community pharmacists often experienced a 【Barrier to medicine counseling】as they would have had to provide (Medication counseling without information) to a (Patient who does not want to talk).

*Since there were many things that truly depended on imagining what patients talked about, if patients did not want to talk much. Even if I asked about the intention of the prescription change, they just said “I’m OK, I know”.*

#### (community pharmacist a)

Even when the community pharmacists had the opportunity to hear to the story of a (Patient who cannot be honest with their doctor), information-sharing was not possible because the doctor had a policy of (Minimal instruction acceptance) by phone. Meetings with the hospital were experienced as (One-way meetings).

Because of (Sparse involvement with hospitals), pharmacists felt that it was difficult to talk to the (Unapproachable university hospital) even when there were problems. In this way they had the【Mindset of being the hospital’s subcontractor】.

*I think it’s probably a personal idea, but it feels like I am a subcontractor. So by any means, I think it’s very hard for me to think about that equality. (Community Pharmacist B).*

Against this background, a【hospital pharmacist’s visit】was facilitated.

*And then, for the first time, when the hospital pharmacist came, I was very surprised. And it happened that they asked us for a collaboration on inhalation guidance ... (Community Pharmacist C).*

The visit triggered 【The beginning of the medical collaboration】.

*It’s nice to hear from the hospital pharmacist, “Let’s all work together and do our best,” and we also feel a sense of intimacy. In that case, it will be easy to say anything, and I think that it is possible to go a little further and for this to become a very good relationship so that it is easy to talk at study meetings as well. (Community Pharmacist D).*

They experienced a 【Confrontation with blood test data】, which was the result of their lack of experience in medical management using examination results.

*Certainly, because we have not learned about blood test values at school, we have to study on our own. (Community Pharmacist E).*

Community pharmacists were 【Puzzled by the tracing report】as they did not know what to report, they expected (Difficulty with document transmission) and questioned the (Speed of doubt inquiry) by faxing the tracing report, rather than contacting the doctors telephonically.

*Hospital pharmacies and doctors say “Please send me a tracing report if you notice something,” but I wonder how much they really want? (Community Pharmacist F).*

By participating in the meetings during the collaboration, community pharmacists felt (Friendship with hospital) and deepened their understanding of the collaboration by the【Recognition of roles】.

*At the meetings I can listen to the doctor’s lecture about what I normally see in the prescription. Since the hospital pharmacy has engaged in such collaboration, I have not quite figured out what I am doing at the hospital yet, but I think that it will become quite clear. (Community Pharmacist G).*

【Interaction with doctors】took place at the meetings, increasing community pharmacists’ familiarity with the doctors. It became easier to consult with doctors by increasing their (Understanding of prescription intention).

*I told a kidney specialist after a meeting, “If I doubt the medication dose, I am somewhat hesitant to ask questions. Because even if there is a problem with the kidneys, I would think that you probably already knew of it, and I would refrain from asking questions.” But doctor said “Oh, please ask me about that kind of doubt; sometimes we make mistakes, even in the kidney specialty.” (Community Pharmacist H).*

*Even the Kyoto University doctor always looks at the name on the prescription, but when I actually feel that the doctor speaks, the meaning is completely different when I look at the prescription, and I feel familiarity. (Community Pharmacist B).*

Community pharmacists developed an 【Awareness of their responsibility】. By checking the blood test values, they could identify an (Indicator of disease condition) and provide an (Accurate medication consultation) for their patient. Their pharmacy became a (Place of OJT).

*I have a sense of awareness that I have to know just because the blood test values have been put on the prescription. And I also feel that some of the medicines that should be more used more mindful about kidney function and liver function are something that we must know.(Community Pharmacist I).*

They improved their skills to 【Change to proper administration of medication dose】 and (Confirmation of adverse events using blood test values) by acquiring knowledge.

*Well, when I contact the doctor about blood tests, the doctor definitely changes the dose with regard to the function of the kidneys. (Community Pharmacist J).*

When a community pharmacist reported a patient’s drug adherence to a doctor, the 【Support of the hospital pharmacists】encouraged and guided them. They showed (Willingness to improve skills), but also experienced (Anxiety about future policy change).

*When the contents were unknown, I received a call from the hospital pharmacist. I think that’s good for us. (Community Pharmacist G).*

The community pharmacists gradually implemented 【Active use of the tracing report】. When they noticed a therapeutic problem in a patient, they decided on the best route to (Provide adherence reports to doctor).

*Well, when I interviewed patients, there were sometimes things I doubted, like adverse events. I reported them and hoped that the doctor referred to them at the next patient visit. (Community Pharmacist K).*

**Fig. 1:**
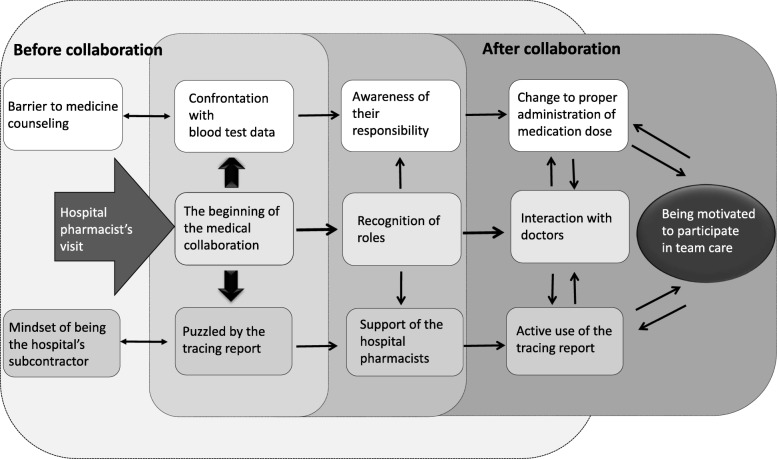
Conceptual Framework of the study Mindset modification of community pharmacist before and after collaboration

Through participation in the collaboration, community pharmacists had more opportunities to be thanked by both physicians and patients, positioning them as a (Pharmacist trusted by patients). This led to them 【Being motivated to participate in team care】(Fig. 1).

*One thing that came directly from the doctor, was “Thank you for providing the information,” which was from the doctor who I gave it to. I thought that was very good. “Oh, I was happy to submit a report!” Already, I was very motivated at that time. (Community Pharmacist K).*

## Discussion

Using the constant comparative method, we analyzed community pharmacists’ change in mindset after increasing their collaboration with university hospital staff in one area of Japan. Triggered by a【Hospital pharmacist’s visit】, Community pharmacists were found to become more conscious of their role as specialists, which led to them【Being motivated to participate in team care】. This was a departure from their previous feelings of inadequacy related to their 【Mindset of being the hospital’s subcontractor】 and their perception that【Pharmaceutical management was difficult】. The direct expression of doctors’ gratitude for information they received often seemed to prompt community pharmacists’ behavioral change. This can be explained using the behavior change model advanced by social cognitive theory (SCT). According to SCT, three main factors affect the likelihood that a person will change a health behavior: (1) self-efficacy, (2) goals, and (3) outcome expectancies. If individuals have a sense of personal agency or self-efficacy, they can change their behavior even when faced with obstacles [13]. Self-efficacy can be defined as a sense of confidence in your ability to take certain actions, and is known to influence thought patterns, behavior, and emotions [14, 15]. Bandura considers self-efficacy the most important personal factor in behavior change, and strategies for increasing self-efficacy include setting incremental goals; behavioral contracting; and monitoring and reinforcement. Reinforcements are responses to behavior that affect whether or not one will repeat it [13]. In this study, community pharmacists received words of appreciation from the doctors in response to their information provision. This contributed to an increased feeling of self-efficacy and reinforced their desire to further participate in the collaboration as they were able to contribute to the treatment of patients. Previous studies have shown that high self-efficacy can help pharmacists improve patient service [16]. The improved patient information-sharing between community pharmacists and doctors helped boost their feelings of self-efficacy, and in turn facilitated the development of their new mindset as experts.

### Beginning of collaboration

Changes in community pharmacists’ mindset about the collaboration began with the【Hospital pharmacist’s visit】. In the interview, one community pharmacist said “I was very surprised that we were being visited by the manager and deputy manager of the Kyoto University Hospital pharmacy.” Before the collaboration began, the community pharmacists generally experienced a【Mindset of being the hospital’s subcontractor】, which is, by definition, an unequal relationship. However, when the hospital pharmacy department initiated the collaboration – particularly the regular meetings – the relationship shifted to become a face-to-face relationship. Snyder, et al. noted that when community pharmacists and physicians begin collaborating, it is important for them to establish a face-to-face relationship, as it can facilitate mutual confidence and clarification of roles [17]. Additionally, previous studies have shown that greater communication and face-to-face relationships between medical staff are more important than knowledge and technical skill to improve the quality of community medical care [18, 19]. In this study, one of the participants reported that forging a face-to-face relationship with the manager of the pharmacy department through multiple collaborative meetings had a positive influence on the participant’s patient care. Overall, this collaboration offered a space – the hospital pharmacy – where community pharmacists, hospital pharmacists, and doctors could forge face-to-face relationships, which in turn improved the ease of information-sharing. Specifically, by sharing the instruction approach for hospital out-patients, hospital pharmacists and doctors guided the community pharmacists to understand what kind of instructions should be given to their patients.

### Process of utilizing the tracing report

The tracing report is a tool used by community pharmacists to inform doctors of issues with a patient’s adherence to their regimen. Medical service fees for tracing reports have been in place since 2012, but these reports are still rarely used [10]. In the early stages of the collaboration, community pharmacists reported feeling 【Puzzled by the tracing report 】 (Table 3). This is evident in one participant’s comment: “I want to tell the hospital doctor about the condition of the patient using the tracing report, but I cannot create the report without knowing what kind of things should be reported”.

In Japan pharmacists are obliged to make pharmaceutical inquiries when doctors issue problematic prescriptions. According to Article 24 of the Pharmacist’s Law, “if there is any suspicion during prescription, consult with the doctor, dentist, or veterinarian who issued the prescription, and after confirming the suspicious point, we must dispense the medication”. Such pharmaceutical inquiries must therefore be done before dispensing, which has the disadvantage of increasing patient waiting time. Meanwhile, according to the Kyoto University Hospital pharmacy, 124 tracing reports were issued in the first eight months of the collaboration – roughly only 0.06% of the total number of prescriptions [10]. This is comparable to the 0.04% found in a national survey in 2014 [9], suggesting that this is a nationwide occurrence. In order to share information with each other using the tracing report, it is necessary for the various role players to improve their 【Recognition of roles】.

### Process of utilizing blood test values

Under the Kyoto University Hospital model, several community pharmacists reported having a 【Confrontation with blood test results】. Some of their comments included: “Only some of the latest blood test data are shared with examination results;” “We have had little experience with examination results so far;” and “It is difficult to manage pharmacies because we do not know the blood test results or treatments of outpatients.” When Joosten, et al. examined the estimated glomerular filtration rate (eGFR) warning system, pharmacists were prompted to suggest a dosage for outpatient prescriptions to the doctor; 66% of these suggestions were accepted [20] This implies that sharing treatment and other medical information, such as blood test results, is indispensable for pharmaceutical management, and that the support of community pharmacists by hospital pharmacists will lead to an improvement in quality of medical care. To progress from their【Confrontation with blood test results】 and ensure that community pharmacists attain an 【Awareness of responsibility 】 – which actually involves referring to blood test results – and reach the point where they 【Change to proper administration of medication doses】, it is essential that the three parties in this collaboration exchange opinions.

### Development of collaboration

This study was conducted in the first year of the collaboration. Although pharmaceutical inquiries related to blood tests and submission of the tracing report were not sufficient in the first year, it is expected that further behavior change will take place as the collaboration continues. According to the transtheoretical model of behavior change, individuals must progress through an initial period (preparation) wherein they determine the opportunities for execution of certain behaviors, after which they become interested in particular behaviors which they ultimately apply (action) [21]. Progression through these phases takes time. The development of a collaborative system between hospitals and community pharmacies is expected to improve medical care quality, including medical safety and patient education, because community pharmacies serve as important medication consultation platforms for many patients [22–24]. In this study, the frequency of meeting participation and number of tracing report submissions varied among the neighboring community pharmacists (Table 2). This suggests that, for pharmacists to contribute more to patients’ treatment, they require a more active attitude toward improving their own skills [16, 24]. At Kyoto University Hospital, it is expected that the collaboration will further develop through community pharmacies’ continuous participation in hospital pharmacy-based initiatives. Furthermore, when other regions adopt the separation of prescribing and dispensing medicine system, the Kyoto University Hospital model can serve as a good guideline. Medical collaboration with community pharmacies can be regarded as a matter of “community organization” [24], and hospital doctors and pharmacists can be said to play a role in empowering community pharmacists. In this way, we believe that collaboration will enable smoother functioning of community pharmacies and will contribute to the treatment of outpatients. However, to maximize these effects, it is important to create a collaborative model that is appropriate for each region.

### Study limitation

In this study, only 11 community pharmacies surrounding Kyoto University Hospital were covered; the behavior changes of community pharmacists in response to collaboration might differ in other areas. Furthermore, since this study was conducted in the first year of collaboration, only the short-term changes could be captured. In addition, purposive sampling was used; while at least one pharmacist from all 11 neighboring community pharmacies was recruited to prevent unbalanced opinions, it is still possible that selection bias occurred. To apply this research to the creation of collaborations between hospitals and community pharmacies in other environments, it is necessary to carefully consider the specific circumstances of those environments.

## Conclusion

Under the Kyoto University Hospital Model, hospital pharmacists encouraged active collaboration between physicians, hospital pharmacies, and community pharmacists by cultivating a face-to-face relationship. This in turn helped community pharmacists become more conscious of their expert status, and thereby participate actively in the treatment of patients.

## Data Availability

The datasets generated during and analyzed during the current study are not publicly available due to privacy protection, but are available from the corresponding author on reasonable request.

## Abbreviations

M-GTA: Modified Grounded Theory Approach
PMDA: Pharmaceuticals and Medical Devices Agency
SCT: Social Cognitive Theory
